# Exploring PFGE for Detecting Large Plasmids in *Campylobacter jejuni* and *Campylobacter coli* Isolated from Various Retail Meats

**DOI:** 10.3390/pathogens3040833

**Published:** 2014-10-21

**Authors:** Daya Marasini, Mohamed K. Fakhr

**Affiliations:** Department of Biological Science, The University of Tulsa, Tulsa, OK 74104, USA; E-Mail: Daya-marasini@utulsa.edu

**Keywords:** *Campylobacter*, plasmids, large plasmids, PFGE, retail meats, mega plasmids, plasmid isolation, alkaline lysis, beef, poultry

## Abstract

*Campylobacter* spp. is one of the most prevalent bacterial pathogens in retail meat, particularly poultry, and is a leading cause of diarrhea in humans. Studies related to *Campylobacter* large plasmids are limited in the literature possibly due to difficulty in isolating them using available alkaline lysis methods. The objectives of this study were to determine the prevalence of plasmids, particularly large ones, in *Campylobacter* spp. isolated from various Oklahoma retail meats, and to explore PFGE (Pulsed Field Gel Electrophoresis) as a tool in facilitating the detection of these plasmids. One hundred and eighty nine strains (94 *Campylobacter* *jejuni* and 95 *Campylobacter coli*) were screened for the presence of plasmids using both alkaline lysis and PFGE. Plasmids were detected in 119/189 (63%) using both methods. Most of the plasmids detected by alkaline lysis were smaller than 90 kb and only three were larger than 90 kb. Plasmids over 70 kb in size were detected in 33 more strains by PFGE of which 11 strains contained larger than 90 kb plasmids. Plasmids were more prevalent in *Campylobacter coli* (73.5%) than in *Campylobacter jejuni* (52%). *Bgl*II restriction analysis of plasmids isolated from 102 isolates revealed 42 different restriction patterns. In conclusion, PFGE was able to detect large plasmids up to 180 Kb in *Campylobacter* spp. which might have been missed if the alkaline lysis method was solely used. *Campylobacter* spp. isolated from retail meats harbor a diverse population of plasmids with variable sizes. To our knowledge, this is the first study to use PFGE to detect large plasmids in *Campylobacter*.

## 1. Introduction

*Campylobacter* is one of the major causes of diarrheal illness and acute gastroenteritis in developed countries [1,2]. Gullian-Barre symptoms and reactive arthritis are chronic consequences associated with *C. jejuni* infections [3]. Plasmids are often found to be associated with antibiotic resistance. Several studies were reported on plasmids of other foodborne pathogens like *E. coli* and *Salmonella* [4]. While a large number of plasmids have been studied and sequenced in *E. coli* [5], very few studies are available to date on plasmids of *Campylobacter* and their possible roles in the fitness of this important foodborne pathogen.

In a study done by Bacon *et al.* in 2000, two plasmids were described in *C. jejuni* strain 81–176 of sizes approximately 45 and 37 kb, named as pTet and pVir plasmids [6]. The pVir plasmid was shown to have a role in invasion [6] and was found to contain 54 ORFs, 35 of them were found to encode *Campylobacter* specific genes [7]. The association between the presence of the pVir plasmid in a *C.* *jejuni* strain and bloody diarrhea is controversial since few studies in the literature seem to be contradictory in this regard. In one of the studies, patients who had the infection with the pVir-positive *C. jejuni* strains were found more likely to produce bloody stool [8] but in another study, the prevalence of pVir plasmids was very low in *C. jejuni* strains isolated from patients with bloody diarrhea despite the presence of plasmids other than pVir [9]. A third study found no association between the presence of pVir plasmids in *C. jejuni* strains and the occurrence of bloody diarrhea [10].

Plasmids in *C. jejuni* and *C. coli* were mostly found to range from 2 kb–162 kb in size, of which plasmids of sizes from 40 kb –100 kb were found to transfer tetracycline resistance via conjugation [11]. Plasmids of size up to 208 kb were documented in a study conducted in Taiwan [12]. Strains with up to 14 plasmids were also found in a *Campylobacter coli* from sheep [13]. Some strains of *Campylobacter* were also found to harbor kanamycin along with tetracycline resistance plasmids [14]. Similarly, *Campylobacter* was found to carry plasmids associated with resistances to gentamycin, penicillin G, and ampicillin [15]. *Campylobacter* species isolated from retail meats were found to be resistant to several antimicrobials like tetracycline, doxycycline, erythromycin, nalidixic acid, and ciprofloxacin [16]. The tetracycline resistance is highly related to the presence of the plasmid-borne *tet*(*O*) gene in *Campylobacter jejuni* [17]. The complete sequence of two large tetracycline resistance plasmids pTet and pCC31 carrying the *tet*(*O*) genes was published more than a decade ago [18].

The prevalence of plasmids in *Campylobacter* differs between clinical and retail meat samples. In a study done by Lee *et al*. in 1994, the prevalence of plasmids in *C. jejuni* was found to be 91% in chicken isolates and 44% in clinical ones [12]. Another study in Germany reported plasmid prevalence of 29% in clinical isolates of *C. jejuni* [9]. Plasmids were detected in 4.5% of *C. jejuni* strains isolated from sheep and 27% of *C. coli* strains isolated from rhesus monkey, swine and poultry [19]. Environmental strains of *C. jejuni* *and C. coli* were found to contain plasmids at percentages of 60% and 50% respectively [20]. Thirty-two percent of *Campylobacter* isolates from the Seattle County Department of Public health were found to harbor plasmids [11]. As it is clear from the above mentioned studies, prevalence of plasmids in *Campylobacter* varies by species, host, meat source, or the location of the study.

Most of the plasmids isolation techniques are based on the alkaline lysis of the cells. The use of Pulsed Field Gel Electrophoresis (PFGE) is helpful in detecting the presence of the large sized mega plasmids by the use of S1 Nuclease [21]. The S1 Nuclease-PFGE is a good method for the screening of mega plasmids with sizes above 100 kb. Large plasmids can be sheared easily and are hard to separate from chromosomal DNA. In PFGE, the cells are lysed within the agarose plugs so that there is less probability for the shearing of plasmid DNA [21]. The literature is lacking, in particular, studies related to *Campylobacter* large plasmids possibly due to difficulty in isolating them using available alkaline lysis methods and the fastidious nature of *Campylobacter*. One of the objectives of this study was to determine the prevalence of plasmids in *Campylobacter* *jejuni* and *Campylobacter coli* strains isolated from various Oklahoma retail meats. Of an equal importance was to explore PFGE as a tool in detecting large plasmids that would have been otherwise missed if the alkaline lysis method was solely used.

## 2. Results and Discussion

### 2.1. Prevalence of Plasmids by Alkaline Lysis and PFGE

A total of 189 isolate (94 *Campylobacter jejuni* and 95 *Campylobacter coli*) which were previously isolated from Oklahoma retail meat samples [22,23] were used for plasmid screening in this study by both alkaline lysis and PFGE. Out of these isolates, 39 isolates were from chicken, 98 from chicken livers, 13 from chicken gizzards, 30 from beef livers, seven from turkey, and two isolates were from pork (Table 1).

**Table 1:** Prevalence of plasmids in *Campylobacter jejuni* and *Campylobacter coli* isolated from various retail meats by alkaline lysis and Pulsed Field Gel Electrophoresis (PFGE).

Meat Sources	No of Strains in which Plasmids Were Detected n/N (%)					
	Alkaline Lysis			Overall (Alkaline Lysis + PFGE)		
	*C. coli*	*C. jejuni*	Total	*C. coli*	*C. jejuni*	Total
**Chicken**	8/9 (89%)	11/30 (37%)	19/39 (49%)	9/9 (100%)	16/30 (53%)	25/39 (64%)
**Chicken Livers**	35/60 (58%)	20/38 (53%)	55/98 (56%)	41/60 (68%)	23/38 (60.5%)	64/98 (65%)
**Chicken Gizzards**	3/3 (100%)	3/10 (30%)	6/13 (46%)	3/3 (100%)	3/10 (30%)	6/13 (46%)
**Beef Livers**	12/19 (63%)	4/11 (36%)	16/30 (53%)	14/19 (74%)	4/11 (36%)	18/30 (60%)
**Turkey**	1/2 (50%)	3/5 (60%)	4/7 (57%)	1/2 (50%)	3/5 (60%)	4/7 (57%)
**Pork**	2/2 (100%)	0	2/2 (100%)	2/2 (100%)	0	2/2 (100%)
**Total**	61/95 (64%)	41/94 (44%)	102/189 (54%)	70/95 (73.5%)	49/94 (52%)	119/189 (63%)

**Figure 1:**
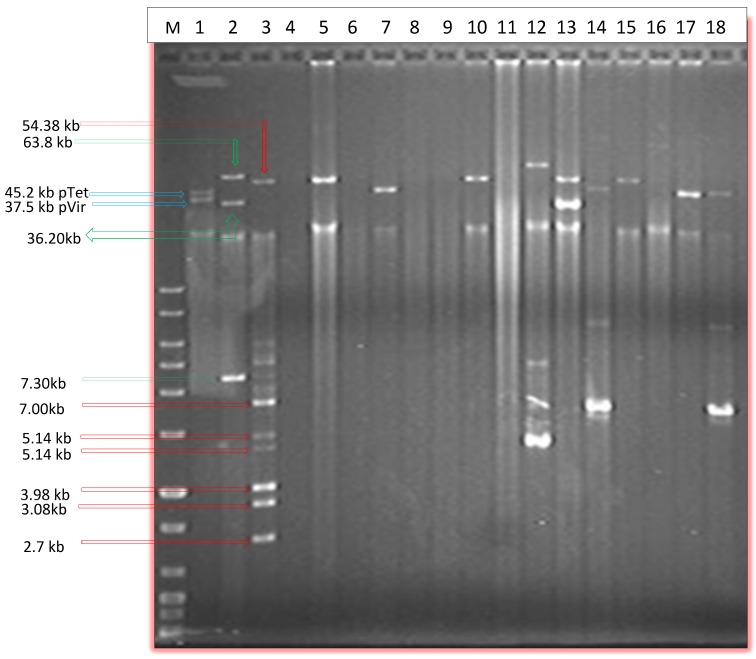
Agarose gel electrophoresis image showing selected plasmids isolated via Qiaprep spin column alkaline lysis method. M, DNA marker; Lane 1, *C. jejuni* 81–176; lane 2, *E. coli* 50192; lane 3, *E. coli* 50193; lanes 4–18 Plasmid mini preps of selected *Campylobacter* isolates.

Plasmids were detected in 119/189 of the screened isolates (63%) using the two plasmid isolation methods (Alkaline lysis and PFGE), while alkaline lysis (Qiaprep Miniprep) alone detecting plasmids in 102 out of 189 isolates (54%) (Table 1 and Figure 1). Prevalence of plasmids was apparently higher in *C. coli* than in *C. jejuni* (Table 1). Approximately 73% of *C. coli* and 52% of *C. jejuni* was found to harbor plasmids by both methods (Table 1). In regards to the source of the retail meat, prevalence of plasmids in *Campylobacter* was higher in chicken (64%) and chicken liver (65%) isolates followed by beef livers (60%), turkey (57%) and then chicken gizzards (46%) (Table 1). Plasmids were also detected in the two pork isolates.

**Figure 2:**
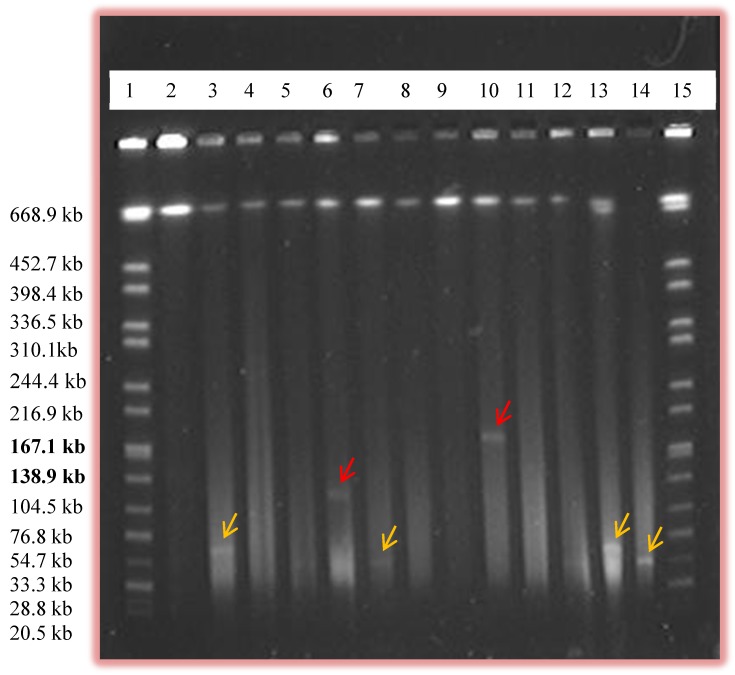
Pulsed Field Gel Electrophoresis (PFGE) gel image showing the presence of large plasmids. Lanes 1 and 15, *Salmonella* serovar Braenderup H9812 marker; lanes 6 and 10 shows the presence of larger plasmids (labeled by red arrows) which were approximately 130 kb and 180 kb, respectively. Other relatively smaller plasmids (labeled by yellow arrows) are also shown in other lanes.

PFGE was able on the other hand to show eight large plasmids (Table 2) above 90 kb in size which were not isolated by Qiaprep or Qiagen plasmid Midi kit (Table 2). Qiaprep was successful in isolating plasmids up to 70 kb in size (data not shown) and three plasmids above 90 kb were also isolated using this method (Table 2). It is worthy to mention that Qiaprep also missed some plasmids ranging in size between 35 kb and 90 kb in addition to the larger ones above 90 kb (Table 2). Some plasmids which were difficult to isolate using alkaline lysis method probably due to their very low copy number being easily detected by PFGE. PFGE was able to detect a plasmid of approximately 180 kb in size (Figure 2).

**Table 2:** Distribution of detected plasmids according to their size in regards to their method of detection (alkaline lysis and Pulsed Field Gel Electrophoresis “PFGE”).

Size of Plasmids (Kb)	# of Plasmids Detected by Alkaline Lysis	# of Plasmids Detected by PFGE
0–45	89	20
45–90	56	81
90–135	3	10
>135	0	1
Total # of Plasmids	148	111

Up to four plasmids were detected on a single isolate in our study (data not shown). In a previous study conducted by Bradbury *et al.* in 1983, the authors showed the presence of 14 plasmids in a single isolate of *Campylobacter* [13]. Although, the distribution of the samples is not even, plasmids in our study were more prevalent in *C. coli* than *C. jejuni* isolated from chicken samples (Table 1). Similarly, more *C. coli* isolates with plasmids were found in beef liver samples than *C. jejuni*. The same was also true in the isolates from chicken livers and chicken gizzards (Table 1). Plasmids were however more prevalent in *C. jejuni* than in *C. coli* isolates from turkey (Table 1).

In our study and following the alkaline lysis method and PFGE, an overall plasmid prevalence of 63% was observed (Table 1). At the species level, plasmids were detected in 73% of *C. coli* and 52% of *C. jejuni* (Table 1). In a similar study conducted by Sagar *et al.* (1987) in Japan, the authors also showed that prevalence of plasmids were more prevalent in *C. coli* than in *C. jejuni* [24]. Similarly, a study conducted by Tenover *et al.* (1985) also showed that plasmids were more prevalent in *C. coli* than in *C. jejuni* [11]. In a study performed on the serologically defined strains of *C. coli* and *C. jejuni* obtained from healthy and diarrheic animals, a little lower prevalence of plasmids were observed [13]. Similar prevalence of plasmids as shown by our Qiaprep method was seen in the clinical isolates of *C. jejuni* isolated from patients with bloody diarrhea [9]. In another study, the overall prevalence of the *C. coli* and C*. jejuni* was also a little lower than ours whereas the prevalence of plasmids in the chicken isolates was a little higher [15]. A Japanese study conducted in 1987 showed a higher prevalence of plasmids in the *C. jejuni* clinical isolates which were tetracycline resistant [24]. Another study performed in Taiwan on *C. jejuni* showed higher plasmid prevalence in chicken isolates than the clinical isolates [12]. Lower prevalence of plasmids was seen in the samples obtained from Seattle-king county public health department [11]. These variable prevalence results are expected due to the variation of *Campylobacter* isolates, their sources, and plasmid isolation protocol followed in each study.

The slightly higher prevalence of plasmids in our study may be due to the use of PFGE which increased the chance of detecting large plasmids and some of the smaller ones missed by alkaline lysis. Thirty-three plasmids larger than 45 kb which were missed by the alkaline lysis method were detected by PFGE (Table 2) which shows the superiority of PFGE in detecting large plasmids.

In our study, PFGE was able to detect plasmids of size up to 180 kb (Table 2 and Figure 2). Here, more plasmids were seen than the number of isolates as some isolates contained multiple plasmids. To our knowledge, our study is the first to use PFGE as a method to screen for the presence of plasmids in *Campylobacter*. The use of PFGE in some other bacteria to screen for plasmids was performed by Barton *et al.* (1994) and plasmids of size up to 244 kb were detected [21]. In our study, some plasmids of sizes approximately 90 kb and above were also detected by alkaline lysis method but PFGE was found to be more efficient in screening for large plasmids. Out of 11 plasmids of sizes above 90 kb, eight of them were seen only by PFGE and were missed by Qiaprep (Table 2). Those mega plasmids were also missed when the Qiagen plasmid Midi kit method was used. In our study, most of the smaller plasmids ran out of the gel during PFGE thus fewer plasmids of size 0–45 kb in size were observed in PFGE compared to Qiaprep (Table 2). On the other hand, 25 plasmids of sizes 45–90 kb, and eight plasmids above 90 kb in size which were missed by Qiaprep were detected by PFGE (Table 2).

While very few studies discussing the presence of large plasmids in *Campylobacter* are available, some have documented the presence of plasmids which were above 100 kb in size. A study conducted by Lee *et al.* (1994) in Taiwan has documented the presence of plasmids up to the size of 208 kb in *Campylobacter* [12]. Similarly, Sagara *et al.*, 1987 has mentioned the presence of plasmids of 135 kb in size in *C. jejuni* isolates, whereas most of the plasmids were found to be of sizes around 45 kb [24]. Tenover *et al.* (1985) have also reported plasmids of sizes up to 162 kb in *Campylobacter* [11]. The majority of plasmid prevalence studies in *Campylobacters* have documented the presence of plasmids from 2 kb–70 kb in size. The fewer reports of the presence of mega plasmids in *Campylobacter* might be due to the fact that researchers use only alkaline lysis methods for plasmid isolation*.* Due to difficulty in growing *Campylobacter* isolates and the probable low copy number of larger plasmids there is higher chances for large plasmids to be missed by alkaline lysis methods. PFGE, while laborious, can be an excellent tool in detecting large plasmids in *Campylobacter*.

### 2.2. Restriction Analysis of the Isolated Plasmids

**Figure 3:**
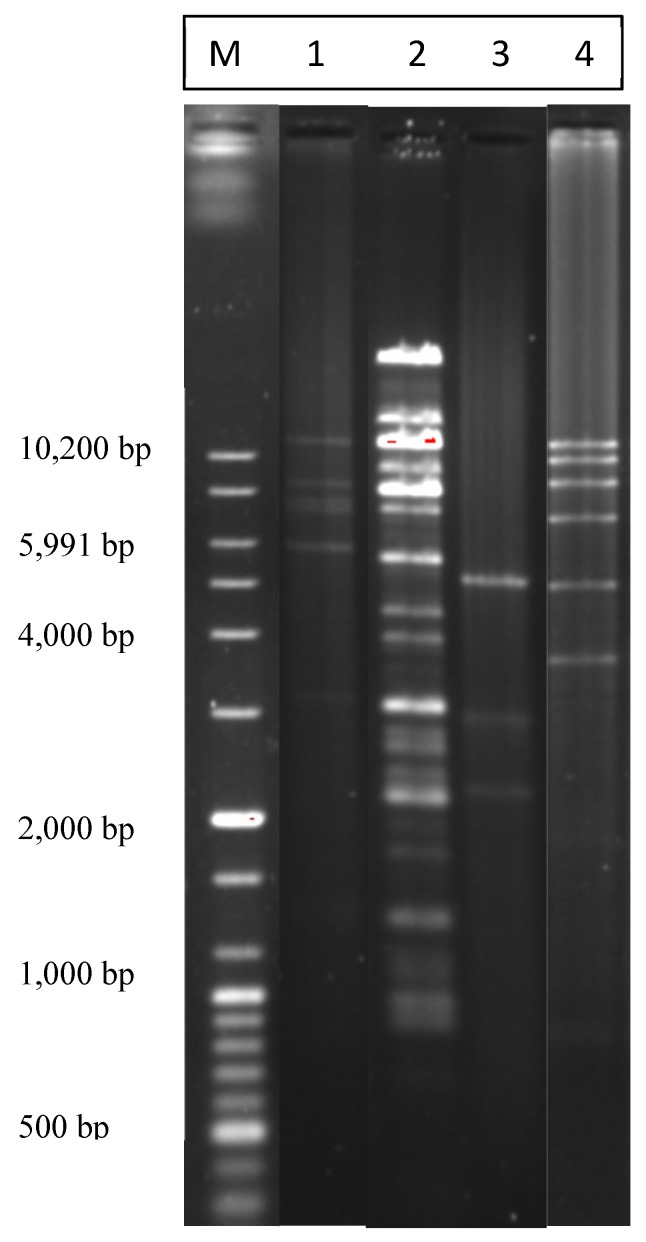
Agarose gel image showing the most common *Bgl*II restriction patterns of plasmids isolated by the alkaline lysis miniprep method. “M”, 100 bp plus DNA ladder marker. “1”, most common pattern; “2”, a pattern for a large plasmid; “3”, the second most common pattern; “4”, one more detected pattern.

Plasmid alkaline lysis mini or medipreps of 102 *Campylobacter* strains were subjected to *Bgl*II restriction digestion (Table 3 and Figure 3). While some plasmid preps showed single plasmids, some others revealed multiple plasmids in the same isolate. A total of 42 different types of restriction patterns were observed (Table 3). Some samples contained more than one plasmid so it was difficult to know the exact pattern of each plasmid separately. Eleven different patterns were shared by more than one strain (Table 3) whereas the rest of the isolates had unique restriction patterns (data not shown). As shown in Table 3 and Figure 3, restriction pattern “A” was the most common pattern detected among the screened isolates (21 isolates). Restriction pattern “C” was the second common pattern (18 isolates), whereas pattern “B” was a restriction pattern for one of the large plasmids in *C. coli* of beef livers (Table 3 and Figure 3). As shown in Table 3, type C, F and H patterns, different species from different sources contained plasmids with the same restriction patterns which might indicate that the two *Campylobacter* species might harbor similar plasmids. Also, the same plasmids can be detected in *Campylobacter* isolated from different retail meat sources.

**Table 3:** Restriction analysis patterns of the isolated plasmids by *Bgl*II.

Pattern	No. of Bands	No of Isolates	Species	Meat Source
A	6	21	*C. coli*	Chicken liver
B	5	5	*C. coli*	Beef liver
**C**	**3**	**4 + 14**	***C. jejuni***	**Chicken + chicken liver**
D	5	3	*C. coli*	Chicken liver
E	4	2	*C. jejuni*	Chicken liver
**F**	**5**	**2 + 2**	***C. coli + C. jejuni***	**Beef liver + chicken liver**
G	6	3	*C. jejuni*	Chicken liver
**H**	**6**	**2**	***C. coli + C. jejuni***	**Beef liver**
I	3	3	*C. coli*	Chicken liver
J	6	2	*C. jejuni*	Turkey
K	2	5	*C. jejuni*	Chicken

Despite the fact that PFGE in our study was able to detect larger plasmids in *Campylobacter* that are up to 180 kb in size, trials to isolate enough high quality DNA from these mega plasmids by alkaline lysis or by electro elution from the PFGE gels were not very successful. This should not deter us from using PFGE to detect large plasmids in *Campylobacter*. We propose here to use PFGE as a screening tool to detect if a particular *Campylobacter* isolate harbors mega plasmids. Once a mega plasmid is detected, the total genomic DNA can be isolated and then subjected to whole genome sequencing using next generation sequencing. The large plasmids sequence can be then separated from the chromosomal DNA sequence after assembly. This whole genome sequencing strategy can help in separating the plasmid sequences from chromosomal sequences as shown in a few recent studies conducted by Pearson *et al.* (2013) which showed the presence of 26,269 bp conjugative cryptic plasmid in *C. coli* clinical isolate, and Chen *et al.* (2013) which showed the presence of a 55 kb gentamycin resistant plasmid in two retail meat *C. coli* isolates using a whole genome sequencing approach [25,26]. Large plasmids detected in our study are currently being sequenced in our laboratory using the Illumina MiSeq next generation sequencing approach.

## 3. Experimental Section

### 3.1. Bacterial Isolates

A total of 189 bacterial isolate (94 *Campylobacter jejuni* and 95 *Campylobacter coli*) previously isolated from Oklahoma retail meat samples [22,23] were used for plasmid isolation in this study by both alkaline lysis and PFGE. Out of these isolates, 39 isolates were from chicken, 98 from chicken livers, 13 from chicken gizzards, 30 from beef livers, seven from turkey, and two isolates were from pork [22,23].

### 3.2. Plasmid Isolation and Screening by Alkaline Lysis and PFGE

Plasmids were first isolated using plasmid spin Mini-preparation column (Qiaprep Miniprep, Qiagen Inc., Valencia, CA, USA). For plasmid Mini-preparation, *Campylobacter* isolates were grown in 5 mL MH broth (MH, Difco, Sparks, MD, USA) with 5% laked horse blood (Hemostat laboratories, Dixon, CA, USA) in a shaking incubator at 200 rpm (Lab-line Orbit Shaker) for 48 h microaerobically at 42 °C in microaerophilic boxes with Pack-Microaero packets (Mitsubishi Gas Chemical Inc., New York, NY, USA). Cells were then harvested by centrifugation, re-suspended and lysed by the reagents provided by the Qiagen kit (Qiagen Inc., Valencia, CA, USA) according to the manufacturer instructions and purified by the provided silica columns. Once plasmid DNA was isolated they were further analyzed by running them on 0.8% Agarose gel at 120 V for 2 h and 45 min and then stained with Ethidium Bromide (50 µL of stock solution in 500 mL of water). Gel images were taken using a Bio-Rad Gel Doc™ XR UV gel documentation system (Bio-Rad, Hercules, CA, USA). For the approximate sizing of the isolated plasmids, plasmid preps of *E. coli* strains NCTC 50192 and *E. coli* NCTC 50,193 along with the 100 bp plus DNA ladder (Bioneer corporation, Alameda, CA, USA) were used as markers.

Further screening of large plasmids was done by Pulsed Field Gel Electrophoresis (PFGE) with the slight modification of the protocol described previously by Barton *et al.* (1994) [21]. Here, 2 µL of a 1:10 unit dilution of 85 unit/µL S1 Nuclease (Promega, Madison, WI, USA) was used for the linearization of circular plasmids [21]. The PFGE plugs were prepared according to the Pulse Net protocol available at CDC website and run under the conditions established by CDC [27] and as detailed previously [28]. First, the isolates were grown on Muller Hinton Agar (MHA, Difco, Sparks, MD, USA) plates with 5% leaked horse blood (Hemostat laboratories) and incubated microaerobically at 42 °C for 48 h. Then, cells were re-suspended in 0.85% NaCl solution and the concentration was adjusted using spectrophotometer (OD between 0.57 and 0.68). The adjusted cell suspension was then supplemented with Proteinase K (Amresco, solon, OH, USA) solution and mixed with equal amount of 1% Seakem Gold Agarose (Lonza, Allendale, NJ, USA) at 50 °C and then transferred immediately to the plug molds. Once the plugs were solidified they were lysed using cell lysis buffer with Proteinase k solution. After lysis, the plugs were washed twice with pure water at 50 °C and followed by washing three times with Tris EDTA (10 mM, 1 mM EDTA, PH-8.0) at 50 °C with constant shaking. Finally, the plugs were stored in cold TE (Amresco, Solon, OH, USA) solution until used [28]. Once the plugs were prepared a small thin slice of the plugs were cut and digested with S1 nuclease (17 units of enzyme/plug) for 45 minutes at 37 °C. Finally, plugs were inserted into the wells of 1% Seakem Gold Agarose and run with the *Salmonella* serovar Braenderup H9812 marker digested with *Xba*I enzyme (Promega, Madison, WI, USA). The gel was then placed into the PFGE apparatus CHEF Mapper PFGE system (Bio-Rad, Hercules, CA, USA) and run for 16 h in 0.5 X TBE (Amresco, Solon, OH, USA) according to the running conditions set by Pulse Net protocol [27,28].

### 3.3. Restriction Analysis of the Isolated Plasmids

Qiagen plasmid midi kit (Qiagen Inc., Valencia, CA, USA) was used to isolate enough DNA of the larger plasmids (above 90 kb in size) following the manufacturer’s protocol. The isolated plasmids by alkaline lysis methods were digested with *Bgl*II enzyme (Promega, Madison, WI, USA). The digestion was done using 5 units of enzyme in 20 µL of reaction followed by incubation for 3 h at 37 °C. Finally, the digestion patterns were analyzed by running on 0.8% agarose gel at 120 V for 2 h and 45 min and finally stained with Ethidium Bromide (50 µL of stock solution in 500 mL of water). Gel images were taken using a Bio-Rad Gel DOC™ XR UV gel documentation system (Bio-Rad, Hercules, CA, USA).

## 4. Conclusions

In this study, 189 *Campylobacter* isolates of various retail meat origins were screened for the presence of plasmids by alkaline lysis and PFGE. Plasmids were more prevalent in *C. coli* than in *C. jejuni* isolates. *Campylobacter* spp. isolated from retail meats harbor a diverse population of plasmids with variable sizes. *Bgl*II restriction analysis of the isolated plasmids revealed 42 different restriction patterns that showed some common patterns among *Campylobacter* spp. from different retail meat sources. PFGE proved to be an effective tool in detecting large plasmids (over 90 kb) in *Campylobacter* which would have been otherwise missed if the alkaline lysis method was solely used. Our data indicate that PFGE is an excellent initial screening tool for the presence of mega plasmids in *Campylobacter*. Once detected, mega plasmids can be isolated if possible, or otherwise sequenced as a part of a whole genome through a next generation sequencing strategy. To our knowledge, this is the first study to use PFGE to detect large plasmids in *Campylobacter* spp.
